# Supplementary material for: An amygdalopontine pathway promotes motor programs of ingestion
Source: bioRxiv. 2025 Jun 8:2025.06.05.657686. Preprint. [Version 1] doi: 10.1101/2025.06.05.657686 (PMC12190466; doi:10.1101/2025.06.05.657686)
Supplement: 1 [file NIHPP2025.06.05.657686V1-supplement-1.pdf]

bioRxiv preprint doi: https://doi.org/10.1101/2025.06.05.657686; this version posted June 8, 2025. The copyright holder for this preprint (which was not certified by peer review) is the author/funder. This article is a US Government work. It is not subject to copyright under 17 USC 105 and is also made available for use under a CC0 license.

Supplemental Figure 1

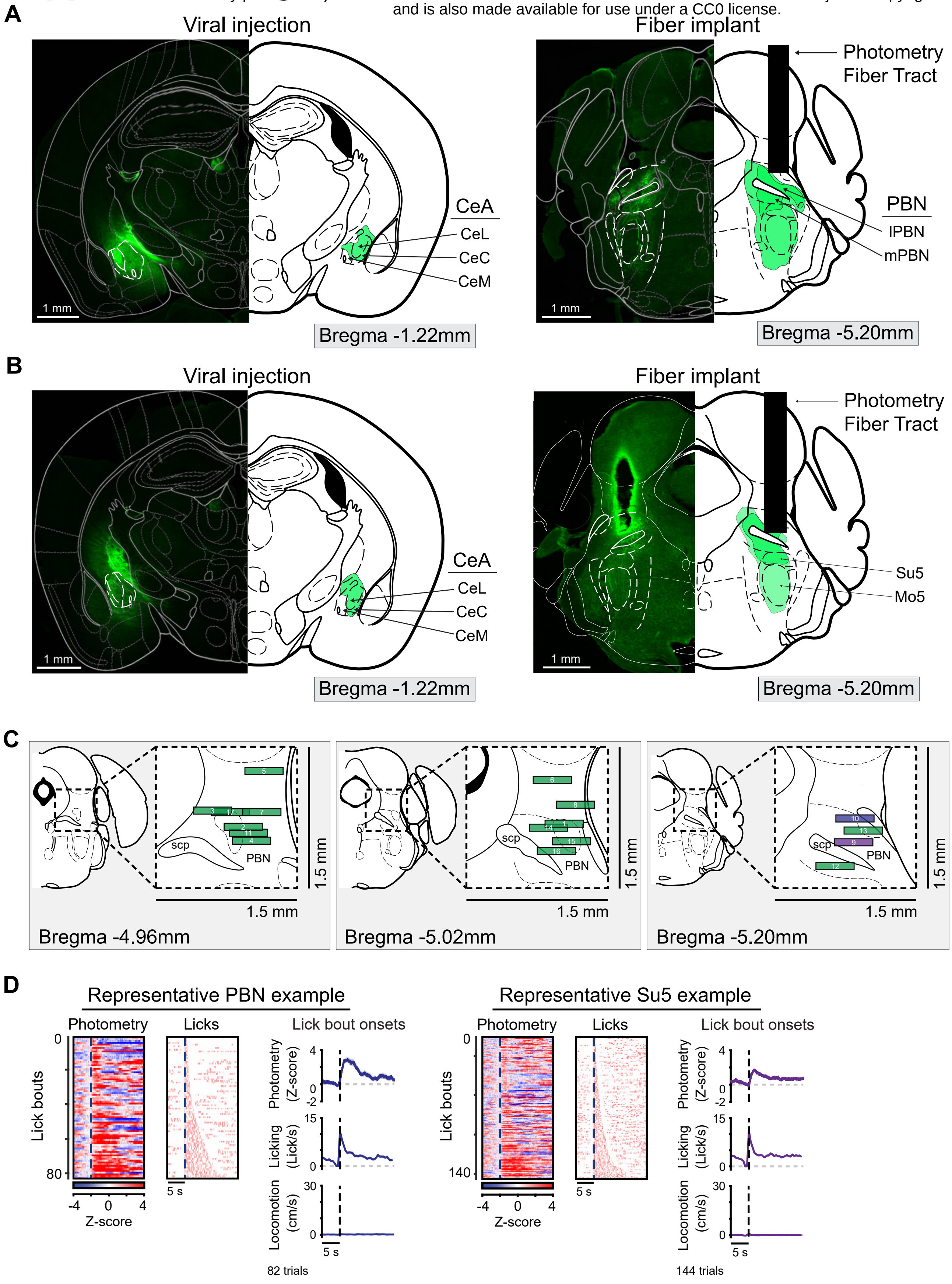

bioRxiv preprint doi: https://doi.org/10.1101/2025.06.05.657686; this version posted June 8, 2025. The copyright holder for this preprint (which was not certified by peer review) is the author/funder. This article is a US Government work. It is not subject to copyright under 17 USC 105 and is also made available for use under a CC0 license.

Supplemental Figure 1: Histology of photometry mice and comparison of signals from probable PBN versus Su5 targets

(A) Left: axon-GCaMP6s expression at the injection site in the CeA. Right: axon-GCaMP6s expressing in axons within dorsolateral pons below the implanted photometry fiber, which was located above the PBN.

(B) Left: axon-GCaMP6s expression at the injection site in the CeA. Right: axon-GCaMP6s expressing in axons within dorsolateral pons below the implanted photometry fiber, which end up implanted through dorsal PBN and likely pick up axonal signals from within mPBN and Su5.

(C) Locations of implanted fibers based on histology from all mice used in photometry experiments.

(D) Left: heatmaps and time courses of photometry, licking and locomotion from a mouse with the fiber implanted above the PBN. Right: heatmaps and time courses of photometry, licking and locomotion from a mouse with the fiber implanted into the PBN and above the Su5.

bioRxiv preprint doi: https://doi.org/10.1101/2025.06.05.657686; this version posted June 8, 2025. The copyright holder for this preprint (which was not certified by peer review) is the author/funder. This article is a US Government work. It is not subject to copyright under 17 USC 105 and is also made available for use under a CC0 license.

Supplemental Figure 2

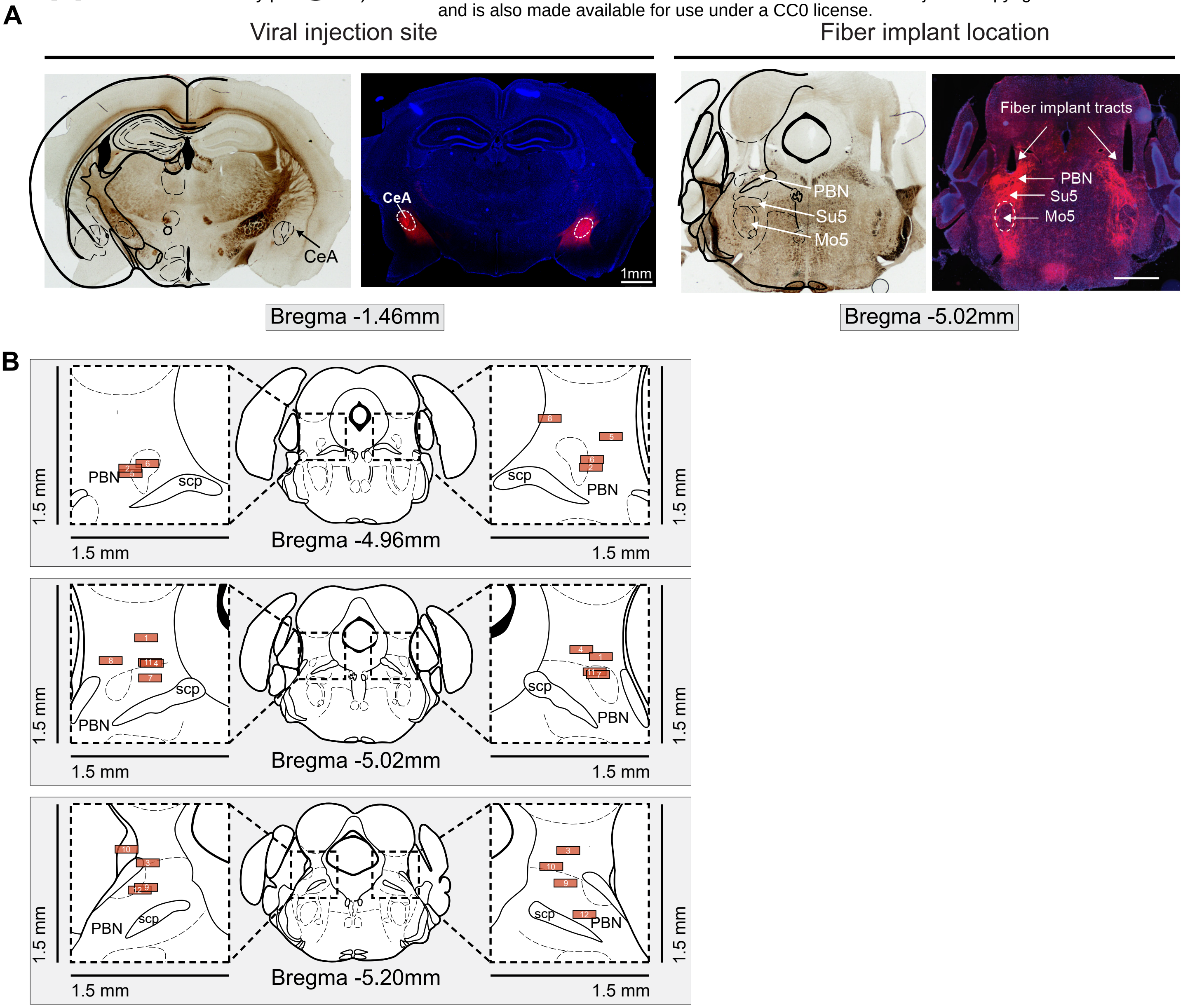

bioRxiv preprint doi: https://doi.org/10.1101/2025.06.05.657686; this version posted June 8, 2025. The copyright holder for this preprint (which was not certified by peer review) is the author/funder. This article is a US Government work. It is not subject to copyright under 17 USC 105 and is also made available for use under a CC0 license.

Supplemental Figure 2: Histology of mice used in the optogenetic studies

(A) Left: Representative brightfield and epifluorescence images depicting bilateral ChrimsonR-tdTomato expression at the CeA injection sites. Right: ChrimsonR-tdTomato-expressing axon terminals in the pons subregions located below the implanted optogenetic fibers.

(B) Locations of implanted fibers based on histology from all mice used in optogenetic experiments.

bioRxiv preprint doi: https://doi.org/10.1101/2025.06.05.657686; this version posted June 8, 2025. The copyright holder for this preprint (which was not certified by peer review) is the author/funder. This article is a US Government work. It is not subject to copyright under 17 USC 105 and is also made available for use under a CC0 license.

Supplemental Figure 3

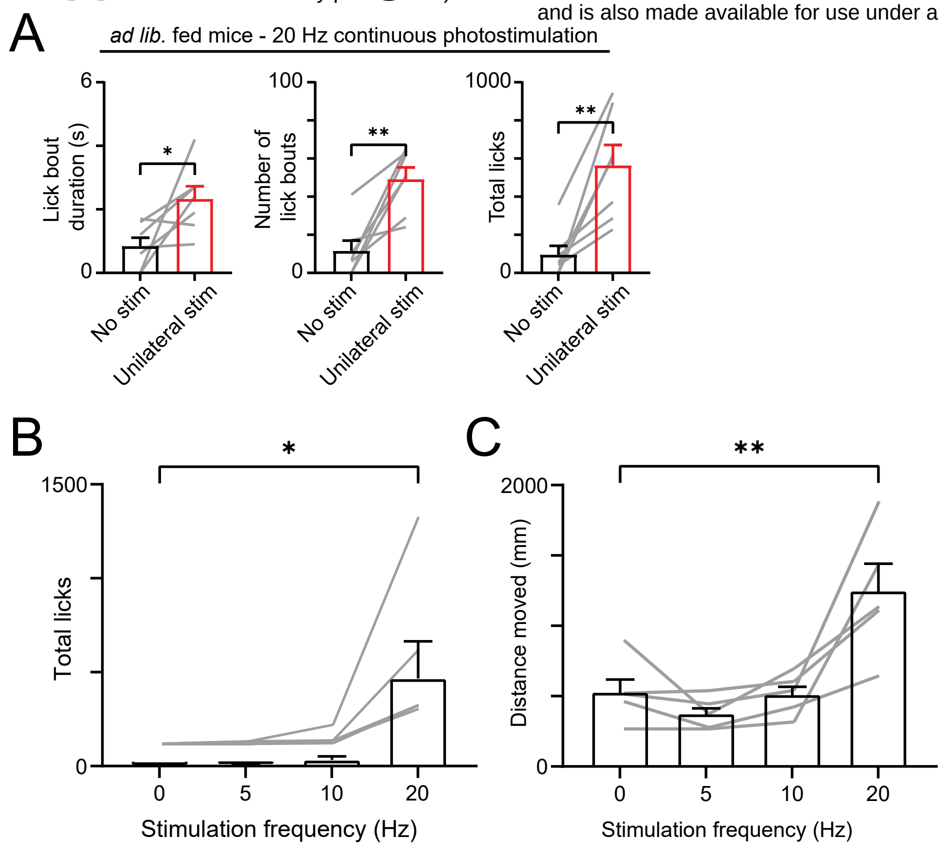

bioRxiv preprint doi: https://doi.org/10.1101/2025.06.05.657686; this version posted June 8, 2025. The copyright holder for this preprint (which was not certified by peer review) is the author/funder. This article is a US Government work. It is not subject to copyright under 17 USC 105 and is also made available for use under a CC0 license.

Supplemental Figure 3: Additional analyses of head-fixed optogenetic experiments

(A) The effect of unilateral 20 Hz continuous stimulation on lick bout duration (left), number of lick bouts initiated (middle), and total number of licks (right) in *ad lib.* fed mice. Two-tailed paired t-test. N = 7 mice (3M, 4F).
(B) Analysis of licks detected by videography in *ad lib.* fed mice during photostimulation of varying frequency. Repeated-measures one-way ANOVA with Dunnett's correction for multiple comparisons. N = 5 mice (3M, 2F).
(C) Analysis of jaw movement distance measured by videography in *ad lib.* fed mice during photostimulation of varying frequency. Jaw movement related to tongue extension is not included in this analysis. Repeated-measures one-way ANOVA with Dunnett's correction for multiple comparisons. N = 5 mice (3M, 2F).

bioRxiv preprint doi: https://doi.org/10.1101/2025.06.05.657686; this version posted June 8, 2025. The copyright holder for this preprint (which was not certified by peer review) is the author/funder. This article is a US Government work. It is not subject to copyright under 17 USC 105 and is also made available for use under a CC0 license.

Supplemental Figure 4

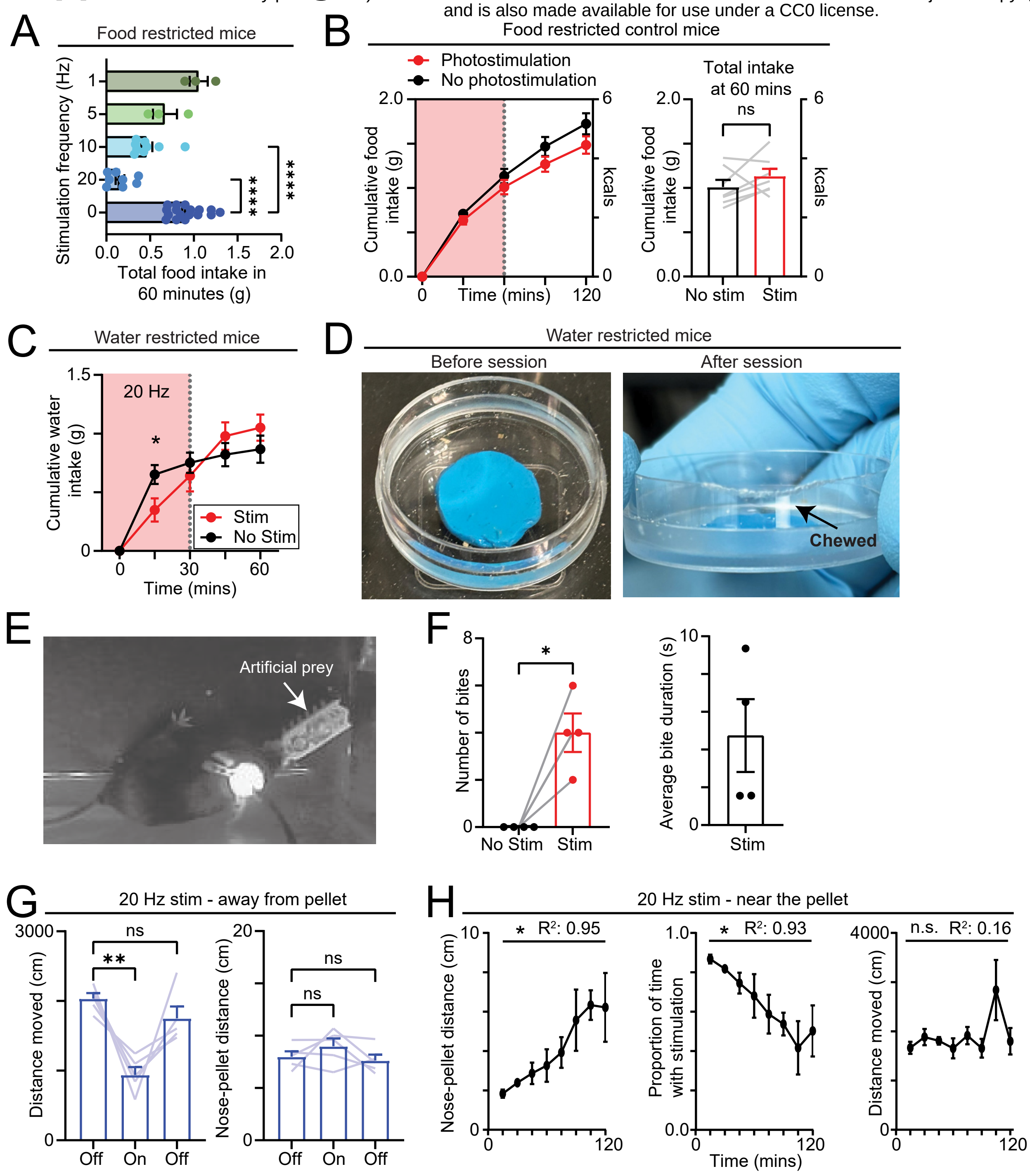

bioRxiv preprint doi: https://doi.org/10.1101/2025.06.05.657686; this version posted June 8, 2025. The copyright holder for this preprint (which was not certified by peer review) is the author/funder. This article is a US Government work. It is not subject to copyright under 17 USC 105 and is also made available for use under a CC0 license.

Supplemental Figure 4: Additional analyses of freely moving optogenetic experiments

(A) Total food consumed in the first 60 minutes without or with photostimulation at different frequencies. Mixed-effects model. N = 17 mice (9M, 8F) without photostimulation, N = 3 mice (3M) with 1 Hz, N = 3 mice (3M) with 5 Hz, N = 9 mice (6M, 3F) with 10 Hz, N = 8 mice (3M, 5F) with 20 Hz.
(B) Left: time course of average food intake of food restricted negative control mice (no Chrimson expression) over 2 hours with either 20 Hz continuous or no photostimulation during the first 60 minutes. Right: total food consumed in the first 60 minutes with or without photostimulation. Two-tailed paired t-test. N = 7 mice (4M, 3F).
(C) Time course of average water intake of water restricted mice over 1 hours with either 20 Hz continuous or no photostimulation during the first 30 minutes. Repeated-measures two-way ANOVA with Šidák's correction for multiple comparisons. N = 5 mice (3M, 2F).
(D) Images depicting 30 mm plastic petri dish used for water intake experiments before and after stimulation from a single example session. We often observed chewing of the petri dish and likely ingestion of the plastic as no plastic debris on cage floor was observed.
(E) Image depicting a mouse biting an artificial prey (toy bug) while receiving 20 Hz photostimulation.
(F) Quantification of the number of bites onto the artificial prey and average bit duration during 20 Hz photostimulation. Two-tailed paired t-test. N = 4 mice (2M, 2F).
(G) Left: distance moved by mice during 20 Hz photostimulation that was triggered if mice were not nearby the food pellet. During the 10 minutes of stimulation (On), mice moved less and were more engaged in fictive eating behaviors. Right: the distance between the nose and the pellet were not significantly different during On or Off stimulation periods.
(H) Left: the distance between the nose and the pellet over the course of 2 hours of close-loop stimulation (20 Hz) just when the mouse was near the food pellet. There was a significant correlation between the nose-pellet distance and time. Middle: the proportion of time during each 15-minute bin that close-loop stimulation (20 Hz) was active. There was a significant correlation between the proportion of time with stimulation and overall time. Right: the distance moved during each 15-minute bin was not correlated with time.

bioRxiv preprint doi: https://doi.org/10.1101/2025.06.05.657686; this version posted June 8, 2025. The copyright holder for this preprint (which was not certified by peer review) is the author/funder. This article is a US Government work. It is not subject to copyright under 17 USC 105 and is also made available for use under a CC0 license.

Supplemental Figure 5

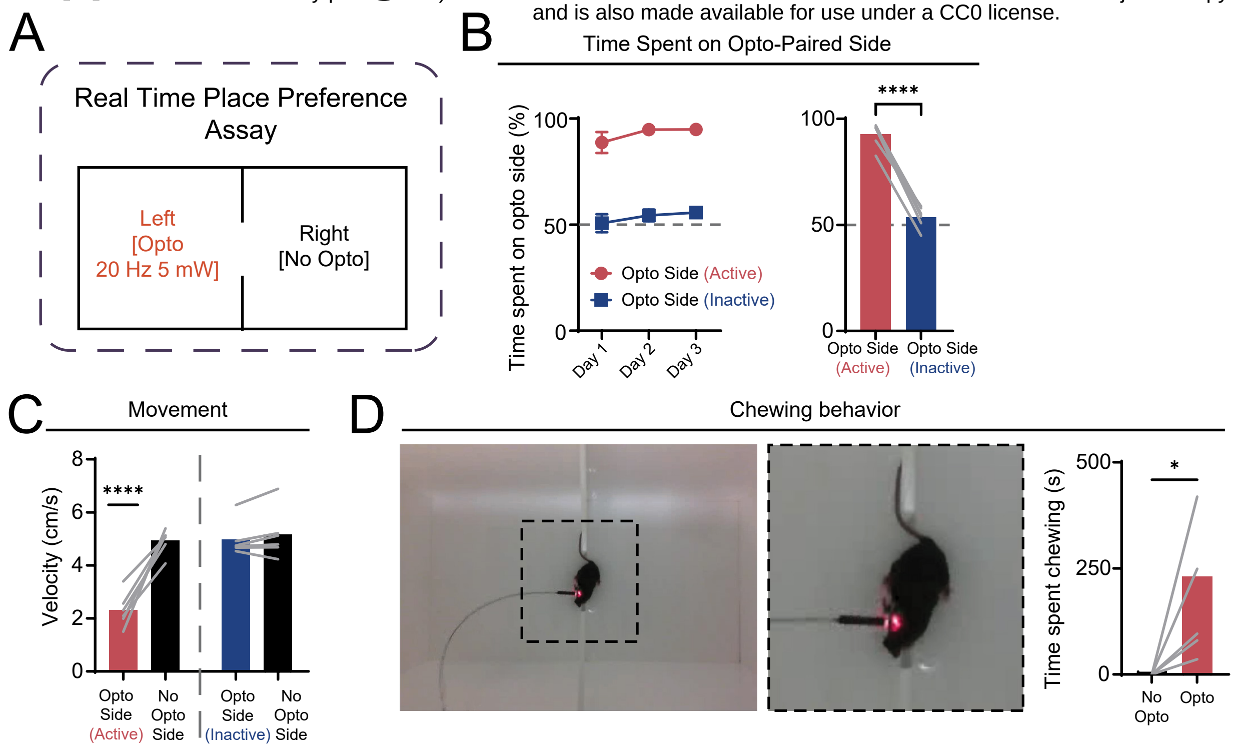

bioRxiv preprint doi: https://doi.org/10.1101/2025.06.05.657686; this version posted June 8, 2025. The copyright holder for this preprint (which was not certified by peer review) is the author/funder. This article is a US Government work. It is not subject to copyright under 17 USC 105 and is also made available for use under a CC0 license.

Supplemental Figure 5: Real-time place preference assay

(A) Schematic of the real-time place preference chamber in which the left side of the chamber delivered continuous 20 Hz (635 nm laser; 5 mW) if the mouse entered that side and persisted until the mouse exited that side of the chamber. Each day involved a 10-minute exploration period during which no photostimulation was delivered on either side of the chamber followed by the 20-minute experiment in which the left side did deliver photostimulation.

(B) Percentage of time spent on the left side of the chamber during 10-minute exploration period in which stimulation was not activated (blue) and the 20-minute period in which photostimulation was active (red). One-sample t-test with a theoretical mean of 50 %. Right: Comparison of the average percentage of time spent on the opto side when the photostimulation was active versus when it was inactive. Two-sample paired t-test.

(C) Movement velocity on each side of the chamber during sessions in which the opto side was active or inactive. Mixed-effects analysis with Tukey's correction for multiple comparisons.

All data: N = 6 mice (4M, 2F).

bioRxiv preprint doi: https://doi.org/10.1101/2025.06.05.657686; this version posted June 8, 2025. The copyright holder for this preprint (which was not certified by peer review) is the author/funder. This article is a US Government work. It is not subject to copyright under 17 USC 105 and is also made available for use under a CC0 license.

SUPPLEMENTAL MOVIE DESCRIPTIONS

Supplemental Movie 1: Tracking of tongue and jaw positions from videography using DeepLabCut analysis

Beginning of movie shows side-by-side views of the same mouse either with no photostimulation (left) or receiving 20 Hz continuous photostimulation (right). The second half of the movie shows the effect of photostimulation on the jaw trajectory from periods of the movie in which no licking is occurring.

Supplemental Movie 2: Example of pellet grasping behavior in head-fixed mice with and without photostimulation of CeA^{pons} axons

Movie shows side-by-side views of the same *ad lib.* fed mouse either with no photostimulation (left) or receiving 20 Hz continuous photostimulation (right).

Supplemental Movie 3: Example of fictive eating behaviors overriding normal pellet ingestion in a food restricted mouse

Movie shows a food restricted mouse either receiving 20 Hz continuous photostimulation and performing fictive eating behaviors instead of eating the available food pellet.

Supplemental Movie 4: Example of CeA^{pons} photostimulation causing biting of a toy bug

Movie top and side views of an example *ad lib.* fed mouse exposed to a toy bug (artificial prey) with no stimulation followed by with 20 Hz continuous photostimulation.

Supplemental Movie 5: Examples of closed-loop CeA^{pons} photostimulation driving food pellet grasping and biting in freely moving mice

Movie shows two examples of an *ad lib.* fed mouse receiving 20 Hz photostimulation only when it approaches and is near food. This stimulation leads to lunging, grasping, biting, and retrieving of the food pellet.

Supplemental Movie 6: Closed-loop CeA^{pons} photostimulation either when the mouse is near food or when it is away from food

Beginning of the movie shows an *ad lib*. fed mouse either receiving 20 Hz photostimulation when the mouse is near food (top) or away from food (bottom). The second part of the movie shows top and side views of an example *ad lib.* fed mouse receiving 20 Hz photostimulation when it is near food for 2 hours (movie is sped by binning to ~3 frames per minute).

Supplemental Movie 7: Examples of fictive eating and cage licking behavior observed during CeA^{pons} photostimulation when the mouse is away from food

Movie shows two examples of fictive eating followed by two examples of cage licking from *ad lib.* fed mice receiving 20 Hz photostimulation when away from food.
